# Computational Elucidation of Structural Basis for Ligand Binding with *Leishmania donovani* Adenosine Kinase

**DOI:** 10.1155/2013/609289

**Published:** 2013-07-24

**Authors:** Rajiv K. Kar, Md. Yousuf Ansari, Priyanka Suryadevara, Bikash R. Sahoo, Ganesh C. Sahoo, Manas R. Dikhit, Pradeep Das

**Affiliations:** ^1^Biomedical Informatics Centre, Rajendra Memorial Research Institute of Medical Science, Patna 800007, India; ^2^Department of Pharmacoinformatics, National Institute of Pharmaceutical Education and Research (NIPER), Hajipur 844102, India

## Abstract

Enzyme adenosine kinase is responsible for phosphorylation of adenosine to AMP and is crucial for parasites which are purine auxotrophs. The present study describes development of robust homology model of *Leishmania donovani* adenosine kinase to forecast interaction phenomenon with inhibitory molecules using structure-based drug designing strategy. Docking calculation using reported organic small molecules and natural products revealed key active site residues such as Arg131 and Asp16 for ligand binding, which is consistent with previous studies. Molecular dynamics simulation of ligand protein complex revealed the importance of hydrogen bonding with active site residues and solvent molecules, which may be crucial for successful development of drug candidates. Precise role of Phe168 residue in the active site was elucidated in this report that provided stability to ligand-protein complex via aromatic-**π** contacts. Overall, the present study is believed to provide valuable information to design a new compound with improved activity for antileishmanial therapeutics development.

## 1. Introduction

Leishmaniasis is a group of vector-borne diseases caused by obligate intramacrophage protozoan parasite of genus *Leishmania*. The global estimates for incidence and prevalence of leishmaniasis cases per year are about 0.5 and 2.5 million, respectively [1]. In India, visceral leishmaniasis (VL) has been reported in parts of West Bengal, Uttar Pradesh, and Bihar. It poses as a major health problem in the state of Bihar, accounting for almost 200,000 deaths occurring solely due to visceral leishmaniasis, which is nearly 90% of the total cases in India (reported since 1977) [2]. Despite the rise in toll for mortality and the deteriorated health conditions, there is no effective treatment available for the cure of this disease. The primary measure for control of leishmaniasis is early diagnosis and its accurate treatment [3]. The available marketed drugs contain many adverse effects and are effective in form of combination chemotherapy only [4]. Thus, it is one of the prime urgency to have an effective and promising therapeutics for this neglected tropical disease [5].

Adenosine kinase (ATP: adenosine 5′-phosphotransferase, EC 2.7.1.20), an enzyme of purine salvage pathway catalyzing the transfer of terminal phosphate from ATP to adenosine, has been shown to possess broad substrate specificity [6]. *Leishmania donovani* lacks the ability to synthesize purines *de novo* and thus is well known to be purine auxotroph [7, 8]. Reports from different authors have revealed the importance of this enzyme in most eukaryotic cells and especially in the purine-auxotrophic parasitic protozoa. Looker and coworkers have reported that the activity profile got provoked almost 50-fold during the transformation of promastigote to amastigote, thereby indicating that adenosine kinase is one of the crucial enzyme targets important for parasite survival [9]. In the year 1987 Datta et al. have reported the isolation and characterization of enzyme in *L. donovani* owing to the importance in parasitology study [10]. Understanding the mechanism of action for this enzyme from *L. donovani* thus can be advantageous in a hope that it may provide relevant information necessary towards designing a novel and specific inhibitor as a therapeutic for leishmaniasis.

Adenosine analogs have advantages for development into antitrypanosomal drugs as reported in a recent study, as many of them, including Ara-A and cordycepin, experimentally proved to pass the blood-brain barrier that is a prerequisite to treat late stage African sleeping sickness [11]. Thus it gives an idea that adenosine analogues can be promising candidates for antitrypanosomal drugs and can be tested against similar targets in other protozoan parasites. A study by Vodnala et al., investigating the trypanosomal adenosine salvage activity by its functional expression in yeast, report a convenient means of testing adenosine antimetabolites for their import and activation [12]. The unique catalytic characteristics of *L. donovani *adenosine kinase (*Ld*Adk) and its stage-specific differential activity pattern have made this enzyme a prospective target for chemotherapeutic manipulation in the purine-auxotrophic parasitic protozoan such as *L. donovani *[13]. Our approach in this study is to find interaction basis of agents that can inhibit *Ld*Adk by *in silico* methods.

The present work aimed at elucidating *Ld*Adk inhibitors by means of computer-aided drug designing, which can guide the rational synthesis of novel inhibitors and may be helpful for developing newer therapeutics. In this report we have prepared a molecular dataset using the reported adenosine kinase (Adk) inhibitors [14–18] and natural products from Nigerian medicinal plants [17]. Since the crystal structure of *Ld*Adk is not reported till date, we proceed with homology modeling. Molecular dynamics (MD) simulation is a well-known technique which has been used here to derive the optimum flexibility of modeled protein structure—*Ld*Adk in solvent medium. Docking calculation has been performed in order to predict inhibitors for *Ld*Adk using different algorithms which is further being validated using MD simulation. 

## 2. Methods

### 2.1. Homology Modeling

Full-length amino acid sequence of *Ld*Adk (accession number: O96439) was retrieved from Universal Protein Resource database (http://www.uniprot.org/) with a predicted molecular mass of 37.14 kDa. The primary amino acid sequence was used to search for a suitable template in Protein Data Bank (PDB) to generate 3D coordinates of *Ld*Adk. NCBI *BLASTp* search was performed against nonredundant database (PDB) with default parameter values [19]. Among the retrieved structural hits, adenosine kinase of *Trypanosoma brucei* (*Tb*Adk) (PDB ID: 3OTX) [20] was found to be the best hit based on maximum sequence identity (53%) and was considered as template for homology modeling. Selectivity of the proposed template in BLAST search was also cross-checked using SWISS-MODEL, RaptorX, CPHmodels *v3.0*, and HHpred [21–24]. Initially ten homology models were generated using Modeller *v9.0 *[23] and one model was generated using SWISS-MODEL web server [19]. The generated models were checked using Discovery Studio *v2.5* from which four models were selected with lowest DOPE score [25]. The models were confirmed using the Verify3D profile analysis method [26]. Stereochemical properties of the models were investigated in Ramachandran plot using PROCHECK analysis [27]. Homology model with maximum number of residues in the favored regions and additional allowed regions in Ramachandran plot was selected for further investigations. The selected model was then refined by loop modeling and side chain refinement to increase the Profile3D score and to make the model reliable [28].

### 2.2. Structure Validation

Structural validation after each loop refinement step was done using ERRAT plot which gives a measure of the structural error for each residue in the protein [29]. The process was iterated until most of the amino acid residues were below 95% cutoff value in ERRAT plot. The refined model was further validated by Verify-3D [28]. ProSA was used to evaluate the generated 3D structure model of protein for potential errors [30]. The root mean square deviations (RMSD) between the main chain atoms of the models and templates were calculated by structural superimposition. Reliability of the models was assessed using Superpose program [31].

For further validation regarding accuracy of homology model and methods used to generate the three-dimensional model, a cross-check validation approach was used. In this strategy, the modeled adenosine kinase *Ld*Adk was chosen as template and adenosine kinase of *Tb*Adk sequence was considered query. Modeller (*v*9.0) program was used, and three-dimensional coordinates were generated for *Tb*Adk. The modeled *Tb*Adk structure was superimposed and comparison of the structures was done by means of all-atom RMSD.

### 2.3. Molecular Dataset

In order to elucidate structural and functional relevance in terms of ligand binding and specificity, docking studies were carried out using different programs. Adenosine kinase inhibitors (organic compounds and natural inhibitors) reported in the literature were generated by ChemSketch [14–18]. The generated structures were subjected to DS 2.5 for ligand minimization. We also blindly docked kinase inhibitors available in PubChem database (http://www.ncbi.nlm.nih.gov/pccompound). The molecules that are sourced from natural medicinal plants [32] were docked and analyzed separately. The molecular dataset prepared for the present study was categorized into (a) organic molecules reported as adenosine kinase inhibitors (dataset O) and (b) natural products (dataset N). Ligand preparations as required by various docking programs were executed separately.

### 2.4. MD Simulation and Active Site Prediction of *Ld*Adk

 MD simulations were conducted for the homology model of *Ld*Adk in explicit solvent using the GROMACS *v4.0.3* (the Groningen Machine for Chemical Simulations) package with Gromos43a1 force field parameters [33]. The model was solvated with 13, 597 water molecules (SPC/E water models) [34] in an octahedron box having edges at a distance of 0.9 nm from the molecular periphery. To obtain the neutrality of the system, six Na^+^ ions were added (charge +6.00) to the system. The solvated system was subjected to energy minimization to remove the steric conflicts between atoms of protein and water molecules having a maximum step of 2000 with steepest descent integrator, that converge the energy when the maximum force was smaller than 1000 kJ·mol^−1^·nm^−1^. Energy minimized model was subjected to position-restrained MD with NPT ensemble keeping number of particles (*N*), system pressure (*P*), and temperature (*T*) as constant parameters. This was carried out for 50,000 steps—a total of 100 ps time period. The reference temperature for coupling was 300 K by Berendsen temperature coupling [35], and 1 atm pressure was maintained by Parrinello-Rahman algorithm [36]. SHAKE algorithm [37] was used to restrain the hydrogen bonds with integration step of 2 fs, and the trajectory snapshots were saved at every 1 ps. The final MD was carried out for 3000 ps (3 ns) under Particle Mesh Ewald (PME) [38] electrostatistics in NPT condition. The probable ligand-binding pockets in the refined model of *Ld*Adk were predicted by Q-Site Finder [39].

### 2.5. Docking Calculation

#### 2.5.1. Docking Using Glide *v9.10*

The protein model was processed using the preparation wizard [40–42] and was optimized for hydrogen-bonding network by reorienting the hydroxyl groups as well as amide groups of Asn, Gln and choosing appropriate states of imidazole ring of His residue. Remaining parameters and steps for receptor grid generation and ligand preparation were similar to the methodology of our previous study [43]. A grid for the docking calculation was prepared after selecting the active site residues. The prepared molecular dataset was subjected to virtual screening protocol where an initial screening was done by high throughput virtual screening (HTVS). Resulted top scoring molecules (10%) were subjected to next round of standard precision (SP) docking. The final top 10% hits from SP docking were subjected to extra precision (XP) mode of docking which were analyzed later. Excellent pieces of literature [44, 45] are available describing the relevance of various mode, of docking using Glide and thus are not discussed in detail here. The molecules that are sourced from natural medicinal plants were docked and analyzed separately. The compounds having docking score below the threshold value (−9.0) were selected. The hits as retrieved from SP docking were used for a cross-check docking calculation using other docking programs like FlexX *v*1.13.5 and GOLD *v*5.1. It was suggested that consensus docking/scoring could be valuable to identify a new lead molecule [46]. 

#### 2.5.2. Docking Using FlexX

 All the default parameters as present in FlexX *v1.13.5* [47] were used for carrying out the docking calculation. Resulting top-scoring 6 molecules (O1 to O6) and top 2 (N1 and N2) molecules from datasets O and N, respectively, are reported here. The receptor description file was defined using the nonhydrogen coordinates of the active site. The molecules used for docking were kept in mol2 file format for carrying out the docking studies. 

#### 2.5.3. Docking Using GOLD

 The active site was defined to encompass all the atoms within a 10 Å radius sphere centered over the active site residues, so as to keep the grid axis the same as that of Glide. GOLD *v5.1* [48] was used for performing the docking studies. For each of 10 independent genetic algorithm (GA) runs, a maximum number of 1000 GA operations were performed. The parameters like: operators for crossover, mutation and migration were set to as 100, 100 and 0 respectively. All other parameters were kept as default. 

### 2.6. MD Simulation of Protein-Ligand Complex

 The coordinates of the top hit molecule with *Ld*Adk as obtained from docking were used for MD simulation. Two protein-ligand complexes were taken, one containing the O1 molecule and another containing N1 with protein. The parameters and simulation conditions were similar to the conditions used for modeled *Ld*Adk. Parameterization for the small molecules was done using PRODRG server (http://davapc1.bioch.dundee.ac.uk/prodrg/) [49]. The simulation was continued for a time scale of 3 ns, and the trajectories were saved at 1 ps interval which was later processed for analysis.

## 3. Results and Discussion

### 3.1. Model Structure of *Ld*Adk

**Figure 1:**
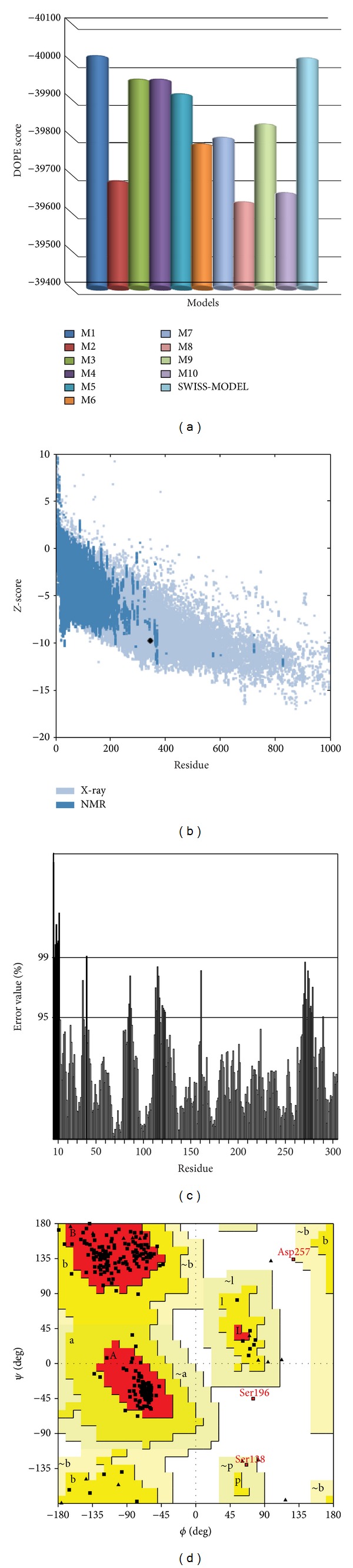
Figures describing various parameters for structure validation. (a) DOPE scores of various homology prepared in the study. (b) *Z*-score plot obtained from ProSA web server. (c) ERRAT plot for residue-wise analysis of homology model. (d) Ramachandran map of the homology model.

 The absence of the crystal structure for *Ld*Adk prompted us to generate a 3D model for docking studies. A high degree of sequence matching (template) is essential for the success of homology modeling. Template search was carried out based on query coverage and sequence identity concept. The BLASTp results for *Ld*Adk showed 53% and 43% sequence identity with the Adenosine kinases of *T. brucei* rhodesiense (3OTX*|*A) and *A. gambiae* (3LOO*|*A), respectively. The template search carried out using various web servers and stand-alone tools also suggested that the crystal structure of *Tb*Adk is the best template: ~53% sequence identity and 73% positives. Different models of *Ld*Adk were built using Modeller and SWISS-MODEL using the best template. Discrete optimized protein energy (DOPE) suggested that the first model generated by Modeller (M1) revealed the lowest energy (−40008.88). The model generated by SWISS-MODEL (S1) also revealed a comparative lower DOPE score (−40003.27) (Figure 1(a)). The other two models, that is, Modeller 3 (M3) and Modeller 4 (M4), also revealed good DOPE scores of −39946.58 and −39946.66, respectively, but are comparatively less than M1 and S1. Upon calculation of Φ/Ψ distribution for backbone conformation, M1 model revealed the highest: 99% residues fell in most favored regions and additional allowed regions and 0.3% residues in generously allowed region, whereas only 0.7% (Ser196 and Asp257) fell in disallowed region (Figure 1(d)). Final structure with lowest energy (M1) was checked by Profile3D (DS *v2.5*). The self-compatibility score for the model appeared to be 162.92, which was much higher than the verify expected high score (157.16) and verify expected lowest score (70.72). All these results suggested the reliability of the proposed model.

### 3.2. Model Validation

**Figure 2:**
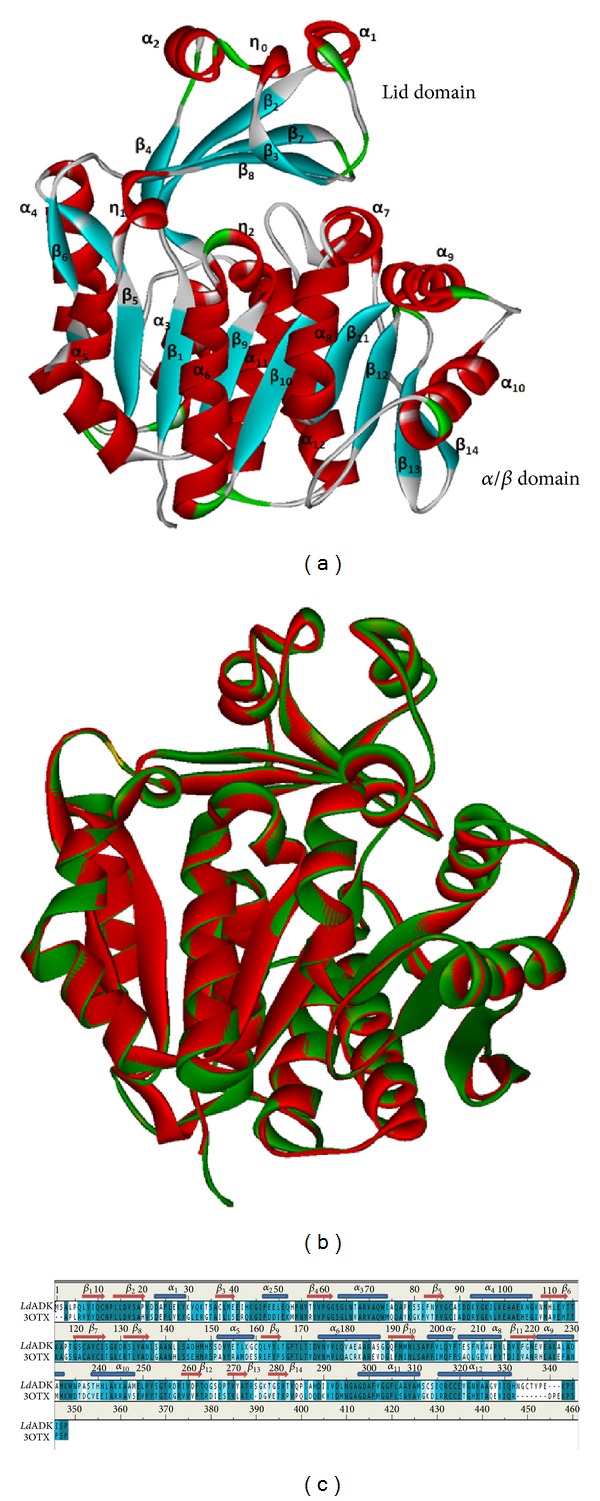
(a) Homology model structure of *Ld*Adk, with the *α*-helix, *β*-sheet, and turn regions shown with different colors. (b) Overlap of model homology structure (red) and crystal structure (green). (c) Sequence alignment window for template (3OTX) and query sequence (*Ld*Adk).

Computer-aided drug design is one of the most efficient tools in the present time, empowering an ease to search novel lead molecules within less span of time with accuracy. In order to proceed for an accurate prediction of novel inhibitors and their interaction profile against *Ld*Adk, it is indeed necessary to have a robust 3D model of the protein structure.  Figure 1 showed the various validation results which help in conferring the rationality of the prepared homology model. Precision of the model was checked using the ProSA server, where the *Z*-score (−9.75) depicted (Figure 1(b)) the model to be within the domicile of reported X-ray crystal structures till date. ProSA web analysis also showed the protein model quality by plotting energies as a function of amino acid sequence position. It demonstrated that the energy remains negative for almost all amino acid residues indicating the acceptability of the predicted model. Similar assumptions were achieved using the ERRAT plot (Figure 1(c)), where the error value of almost all residues for the protein (87.53%) was found below the cutoff limits. Superimposition of *Ld*Adk model with its closest homologue *T. brucei* resulted in a root mean square deviation (RMSD) of 0.467 $\overset{´}{Å}$ (Figure 2). The overall structure of *Ld*Adk contained a mixed fold and consisted of 12 *α*-helices and 14 *β*-strands with two distinct domains (Figure 2(a)). The smaller lid domain appears as *α*/*β* two-layer structure formed by five *β*-sheets (*β*2, *β*3, *β*4, *β*7, and *β*8) and two solvent-exposed *α*-helices (*α*1 and *α*2) located on top. The large domain was *α*/*β* domain composed of nine *β*-sheets (*β*1, *β*5, *β*6, and *β*9–*β*14), of which *β*13 was antiparallel. The *β*-sheets were surrounded by 10 *α*-helices (*α*3–*α*12) exposed to the solvent. A good structural overlapping was noticed between the model and the template, especially in *α*-helices and *β*-sheets regions (Figure 2(c)). The conservation of this sequence and the structural superimposition suggested that the *Ld*Adk model is robust and accurate. In another validation approach the energy-minimized and loop-refined model of *Ld*Adk was used as a template to build the model of *Tb*Adk. This model was used to superimpose with the crystal structure (3OTX). Interestingly we have observed an all-atom RMSD of 0.568 Å, which suggested that our homology model was far more accurate.

### 3.3. MD Simulation of *Ld*Adk Model Structure

**Figure 3:**
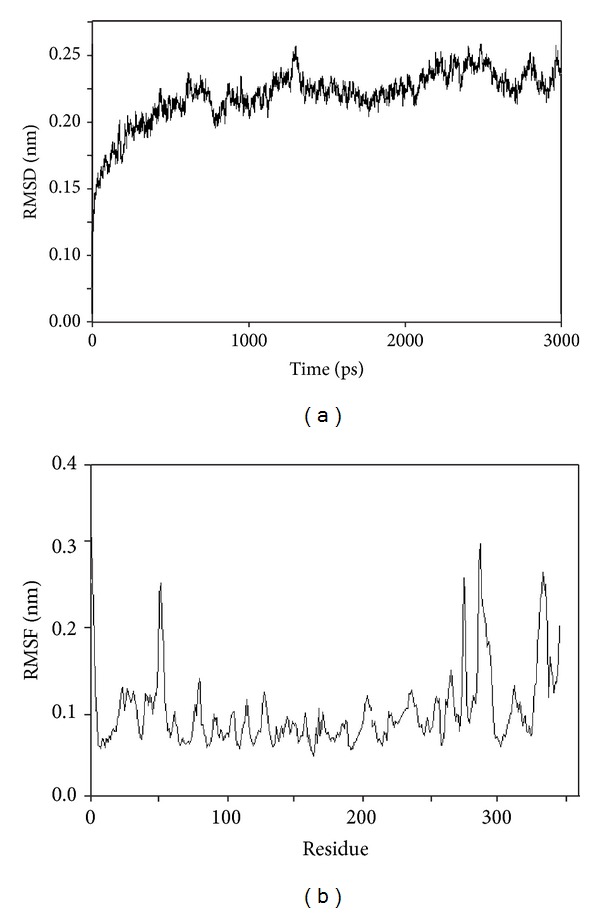
MD simulation results for *Ld*Adk protein model. (a) RMSD plot for the model system. (b) RMSF plot for the model system.

MD simulation is one of the well-known theoretical techniques, popularly used for assessing the stability of any predicted three-dimensional model. The prepared three-dimensional homology model of *Ld*Adk was processed for MD simulation for a 3 ns time scale in the explicit solvent condition.  Figure 3(a) showed all-atom RMSD of the modeled system against the time scale. An initial jump in the RMSD at 0 ps time depicts the minute adjustment of the protein model in the solvent condition. In general, the RMSD of any system, within 1 Å (10^−10^), can be regarded as stable, and the deviation was found to occur within a range of 0.15–0.25 nm (10^−9^) for the proposed model and suggested that the system was having a customary behavior in explicit condition under the effect of force field. Apparently, we also tried to get a depth for dynamicity of each residue by means of root mean square fluctuation (RMSF) (Figure 3(b)). The rmsf plot for the model system depicted similar stability and was well correlated with the RMSD plot. Interestingly, it showed much flexibility of resides in the range of 50–60 and 260–300 regions. According to the active site prediction as discussed in the earlier section, we found the same residues in these regions. Thus, MD simulation data of the model *Ld*Adk illustrated that the active site residues were precisely dynamic as compared to the other residues of the model. As a general observation, for any enzyme, either the active site or the loop facilitating the opening and closing of the active site channels is found to be dynamic, so as to facilitate the catalysis activity. The range of dynamicity of these active site residues fell within a range of 0.1–0.2 nm, suggesting the side chains of these amino acids were flexible.

### 3.4. Docking Calculation

Theoretical validation of the *Ld*Adk model confirmed the accuracy of the three-dimensional structure which can be used for prediction of suitable chemical inhibitory agents. The prepared molecular datasets O and N were used for our docking studies with modeled *Ld*Adk. The predicted active site as discussed in the methodology section was used to dock all the molecules. The initial docking experiment was exercised using Glide module. The obtained molecules from SP mode of docking (part of virtual screening protocol) were used for comparative docking analysis using FlexX and GOLD. The final molecules as conferred from the XP results which were in good agreement with the results of FlexX and GOLD docking were adopted for visual analysis.  Table 1 showed the various docking scores of the top six molecules from dataset O belonging to series of pyrrolo[2,3-d]pyrimidine nucleoside as reported by Ugarkar et al. [15] and top two molecules form dataset N. It was found that molecules O6, O2, and O3 were having Glide score > −11, whereas molecule N1 was having much higher Glide score of −14.5. Such results are much motivating as they generate interest to seek the insight of structural feature which provokes the difference in docking studies.

**Figure 4:**
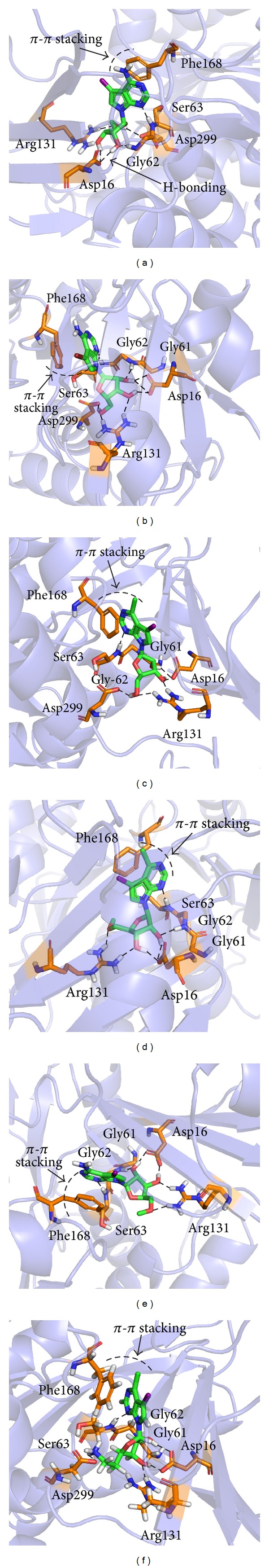
Interaction profile for the organic molecules, from docking studies for molecules (a) O1, (b) O2, (c) O3, (d) O4, (e) O5, and (f) O6.

Figure 4 showed docked pose of six molecules (O1–O6) which showed almost similar kind of docking results with different algorithm as implemented in software. The detailed interactions of the top-scoring molecule were given in Table 2. Hydroxyl groups attached to the 3 and 4 position of tetrahydrofuran were found to form strong hydrogen bonds (H-bond) in all the cases with the side chains of Arg131 and Asp16. The positively charged side chain of Arg131 was making H-bond contact with the electron-rich oxygen atoms of diols whereas to the hydrogen atoms, the negatively charged side chain of Asp16 makes H-bond. It was also evidenced from the docked pose that the amino acids Gly61, Gly62, and Ser63 were interacting with the chemical moieties in all the cases. Asp299 was found to form polar contacts in few cases only (Figures 4(a), 4(b), 4(c), and 4(f)). The interaction with Asp299 can be considered because of larger functional groups attached, such as –CH_2_OH or –C_2_H_2_NH_2_. Interestingly, the energetically most favored contact was found with Phe168 in all the cases which were governed by virtue of *π*-*π* stacking interaction. The fused heterocycle ring pyrrolo[2,3-d]pyrimidine was found to be stacked with the *π*-electron clouds of phenyl ring of Phe168 residue which was believed to provide energetic favor to the molecular conformation. Since the docking protocol utilizes the rigidity in the protein conformation, it is thus essential to get account of the dynamicity for active site residues in association with the flexibility of chemical moieties.

**Figure 5:**
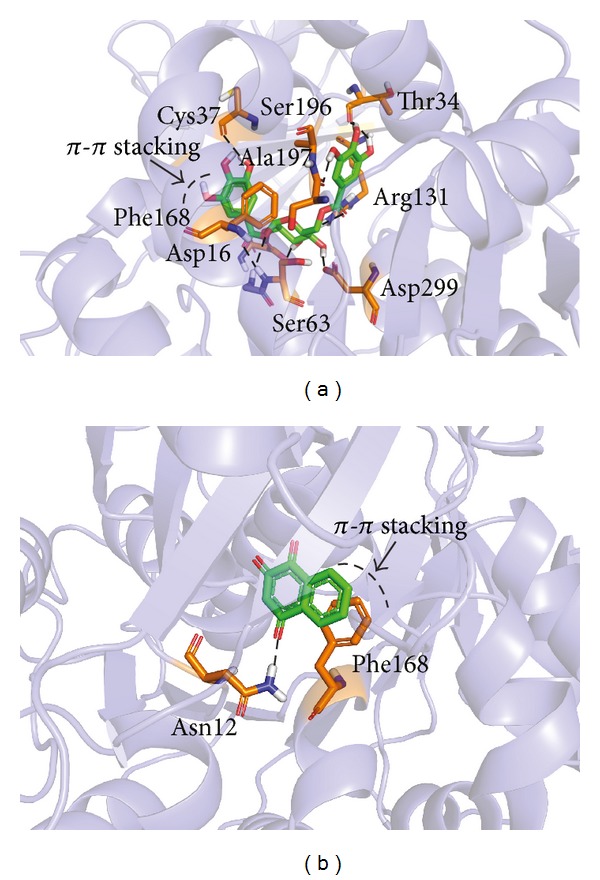
Interaction profile for the natural products, from docking studies for molecules (a) N1 and (b) N2.

Docking results for N1 and N2 from dataset N were presented in Figure 5. Top-scoring hit N1 (1,6-digalloylglucose) was from source *Acacia nilotica*. A similar kind of interaction profile was found in this case which is comparable to the docking results found for O1 to O6 molecules. Active site residues Asp16, Ser63, Asp299, and Arg131 were found to form strong hydrogen bonds with the sugar moiety of N1. Out of these, Asp16 and Asp299 were found to behave as H-bond acceptors, whereas Ser63 and Arg131 were found to behave as H-bond donors while interacting with the sugar moiety. The hydroxyl groups attached to the aromatic ring parts of the molecule were found to be stabilized by virtue of polar contacts with the residues like Ala19, Thr34, Cys37, and Ser197, behaving as H-bond acceptors. The results seemed to be in agreement where the rich *π*-electron cloud of aromatic system, having a pronounced electron-donating effect over the hydroxyl groups, and thus the H-bond formed by the system with the active site residues can be thought to be much stronger. Interestingly, the *π*-*π* stacking interaction was also found to be dominant in this case where the aromatic ring of the molecule was involved in aromatic stacking with the side chain of phenyl ring of residue Phe168. 

 Molecule N2 (lawsone), which was considered for analysis in our study based on the docking calculations, was sourced from *Lawsonia inermis*. N2 (chemical name: 2-hydroxynaphthalene-1,4-dione) has a core naphthalene group. The reason for considering this small phytochemical in our study is its structure, which is easy to adopt for synthesis of series of molecules and can be tested for activity in the future. The docking study of N2 did not provide any remarkable interaction with the amino acids, except the carbonyl group attached to the naphthalene ring at position 4, and was found to make one H-bond with the amine group of Asn12. However, the *π*-*π* stacking interaction was prominent in this case with Phe168, which can be regarded as the key interaction facilitating a stable molecular conformation within the receptor macromolecule. 

### 3.5. MD Simulation of Ligand Molecules with Protein

**Figure 6:**
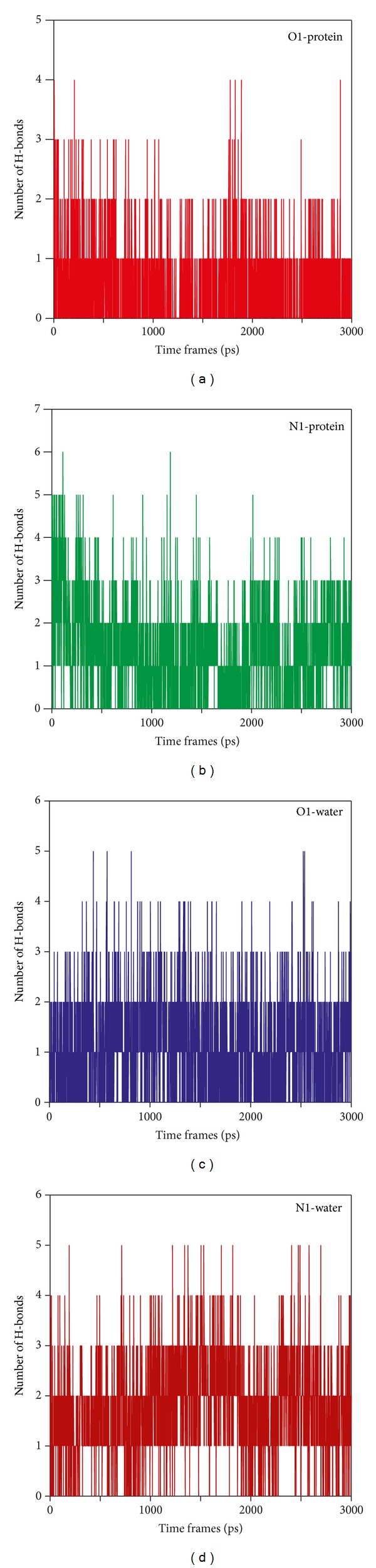
H-bonding profile between molecules and ((a) and (b)) protein and ((c) and (d)) water from MD simulation.

From the results of MD of the modeled protein, we observed that the active site residues were indeed dynamic as compared to the other atoms in the model, whereas docking studies suggested many polar contacts in the active sites which facilitate binding based on the docking scores. Ever since, the protein model considered for the docking calculation was a rigid macromolecule; thus to validate the results we used all-atom MD simulation for ligand-protein complex. As conferred from Figure 3, the RMSD of the protein was much stable; thus we have not assessed the RMSD for the ligand-protein complex. Polar interactions of the system were considered with number of H-bonds formed between protein and ligand in the simulation time course.  Figure 6 depicted the quantitative H-bonding profile for both the O1 as well as the N1. For O1, two H-bonds seemed to provide much stronger contact with the protein (Figure 6(a)), whereas in N1, the same contact was being stabilized by 2-3 strong H-bonds. This gives an assumption that despite the dynamicity of active site residues, the ligand molecules were still intact within the active sites. To further confirm our observation, we checked the polar contacts formed within the ligand and solvent molecules. Since docking calculations have the dielectric effect of the water models as an implicit effect to add up in the scoring scheme, the quantitative measurement of such interaction profile had been taken into account by virtue of MD simulation. In both the cases, we observed almost similar H-bonding profile between the ligand molecules and water models, which ranges from 2 to 3 strong polar contacts. Thus, RMSF fluctuation of model *Ld*Adk and the H-bonding profile of ligand-water suggested possible explanation, what factors make ligands intact within active site. Ever since the flexibility of active site residue is essential for the catalytic activity of any enzyme; this is indeed facilitated by the solvent perturbation inside the physiological condition. Thus, water molecules always show H-bonding network within the active site with the substrate. Similar observation was found in this study where the water molecules formed strong H-bond with the ligand. 

**Figure 7:**
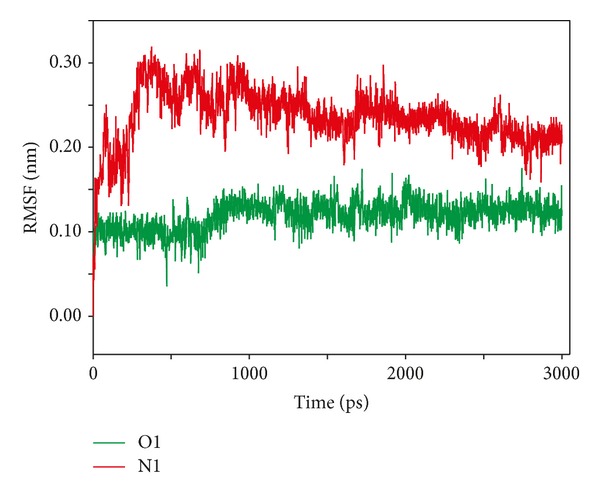
Root mean square fluctuation for the ligand atom plotted against time from MD simulation.

 To confer such results, rmsf of the ligand molecules with the active site in the simulation time period was assessed (Figure 7). An initial fluctuation of the molecules at the starting time scale of simulation conferred to the conformation adjustment due to energy minimization and equilibration prior to the production run. O1 showed no major fluctuations while interacting with the protein macromolecule and thus can be concluded to be much stable. In case of the N1, a shift in the rmsf was found corresponding to the first 500 ps. N1 seemed to be stabilized in the later part of simulation, as the rmsf did not fluctuate till the end. 

**Figure 8:**
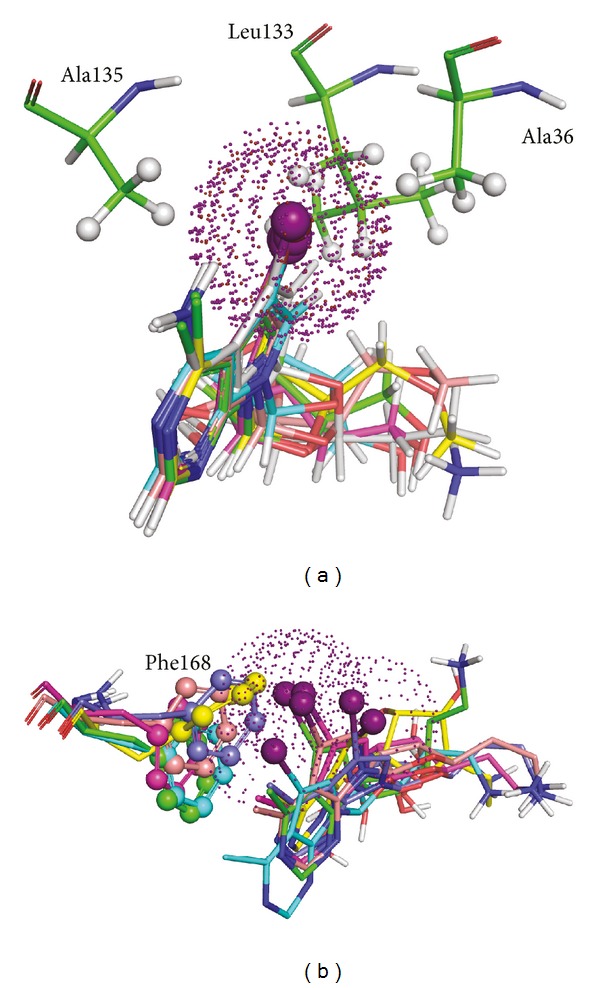
Van der waals and *π*-stacking interactions between halide atoms from organic molecule. (a) Structures from docking studies of all ligands with the nearby amino acid residues. (b) Structures from MD snapshots.

 Another interesting feature observed from the MD simulation and was cross-checked with the docking studies for O1 is the stability of halogen group in ligand.  Figure 8(a) showed the van der Waals region for the halide atoms attached to the pyrrolo[2,3-d]pyrimidine ring with the nearest aminoacids from the docking calculations (interacting atoms areshown in ball and stick representation). Halide atoms were electronegative in nature, and stability of such functional moiety cannot be achieved from the hydrophobic side chains of residues such as Ala36, Leu133, and Ala135. Analyzing the frames of trajectory, a suitable explanation was found for what interaction could accompany electronegative *π*-clouds of halide atoms. MD simulation snapshots taken at an interval of 500 ps revealed that the *π*-cloud of aromatic phenyl ring of Phe168 not only interact with the aromatic *π* electrons of pyrrolo[2,3-d]pyrimidine ring but also accommodates the lone pair *π*-electrons of halide ion to provide stability of the ligand molecule in the active site. These results can explain the initial conformation fluctuation as evidenced in initial timeframe (Figure 8(b)). Thus, the overall MD results suggested a stable interaction between the ligand molecules and the *Ld*Adk protein.

### 3.6. Correlation with Previous Studies

Ghosh and Datta have elucidated presence of arginine residue in active site of *Ld*Adk which is crucial for enzyme activity. Treatment of *Ld*Adk with arginine-specific reagents such as phenylglyoxal, butane-2,3-dione, and cyclohexane-1,2-dione has shown irreversible inactivation of the enzyme [50]. A similar assumption was adopted by Costa and coworkers in which they made an attempt to find out active conformation of chemical moiety *neolignan* sourced from plant family Myristicaceae, which exhibited prominent interaction in the active site of *Ld*Adk [51]. It was assumed that active *neolignans* interact with arginine residue. Interestingly, all the six molecules form dataset O (namely, O1 to O6) and N1 showed prominent polar contact with Arg131 as conferred from our docking studies. In another report Datta et al. have shown that mutation of Arg131 causes significant reduction in catalytic activity of *Ld*Adk [52]. Moreover they also concluded that Asp299 and Arg131 both are key catalytic residues in the protein; similar inference has been highlighted from our docking studies (Figure 4). The diglycyl motif (formed by consecutive glycine residues: Gly61 and Gly62) present in the active site are responsible for maintaining conformation flexibility [52] and thus was thereason the rmsf data from modeled *LdAdk* showed high fluctuation as compared to other residues of the protein. In other reports from the same laboratory, they also established that Asp16 is also one of the crucial residues in the active site and is found in our study also where Asp16 is actively involved in forming H-bonds with the ligands. Thus, it clearly explains that these residues are important to be considered from synthetic and experimental drug design point of view [53]. Remarkably, the importance of Phe168 was explored in our study, which is believed to provide significant contribution in ligand binding to *Ld*Adk. These structural insights from aromatic-*π* interaction may be regarded as one of the key factors in lead design.

## 4. Conclusion

Construction of a robust homology model of *Ld*Adk in combination with docking and molecular dynamics simulation results in valuable structural insights for ligand binding. Importance of arginine and aspartic acid residues for a stable ligand binding was well correlated with the previous of literature. Specific role of phenylalanine residue is highlighted in the present study, which is crucial for designed ligand molecules having aromatic pharmacophores. The said contact is believed to be favorable via aromatic-*π* interaction and can be useful for designing selective inhibitors against *Ld*Adk. Molecular dynamic studies of ligand-protein complex also elucidated that the role of water molecules was also important for stability of ligand moieties in the active site by providing polar contacts. All these pieces of information are believed to be useful for rational design prospective of novel antileishmanial compounds.

## Figures and Tables

**Table 1:** Docking scores of top-hit ligands predicted from different structures.

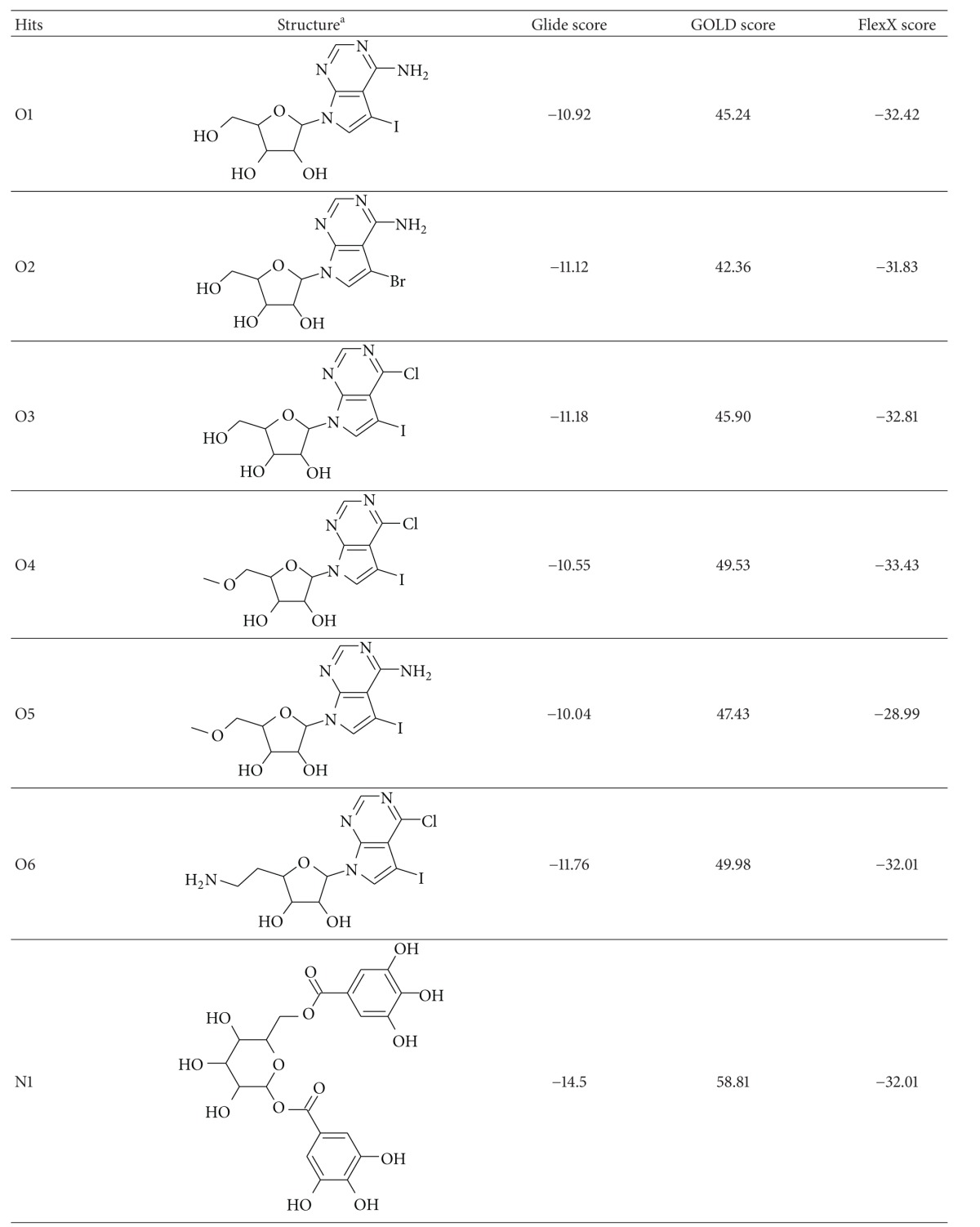 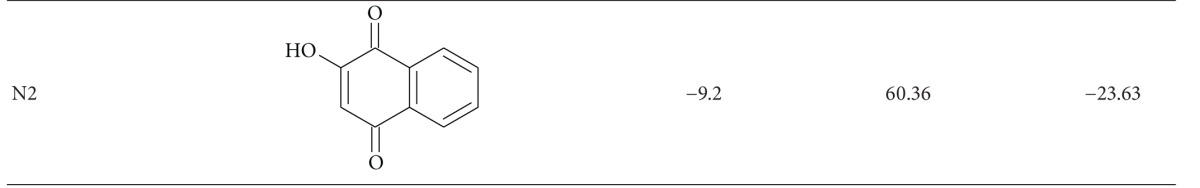

^a^The structures have been generated using ACD/ChemSketch (http://www.acdlabs.com/).

**Table 2:** Polar contacts information from docking calculation between ligands and protein.

Hits	Residue	Atoms	Distance (Å)
O1	Asp 16	O(H)*⋯*(O), O(H)*⋯*(O)	1.8, 2.0
	Gly 62	(O)H*⋯*N(H)	1.7
	Ser 63	(N)*⋯*N(H)	1.7
	Arg 131	(O)H*⋯*N(H), (O)H*⋯*N(H)	1.9, 2.1
	Asp 299	O(H)*⋯*(O)	2.1

O2	Asp 16	O(H)*⋯*(O), O(H)*⋯*(O), O(H)*⋯*(O)	1.9, 2.3, 2.1
	Gly 62	(O)H*⋯*N(H)	1.9
	Ser 63	(N)*⋯*N(H)	2.0
	Arg 131	(O)H*⋯*N(H)	2.1
	Asp 299	(O)H*⋯*N(H), O(H)*⋯*(O)	2.4, 1.6

O3	Asp 16	O(H)*⋯*(O), O(H)*⋯*(O)	1.8, 1.9
	Gly 62	(O)H*⋯*N(H)	1.7
	Ser 63	(N)*⋯*N(H)	1.8
	Arg 131	N(H)*⋯*(O)H, N(H)*⋯*(O)H	1.7, 2.2
	Asp 299	H(O)*⋯*(O)	2.0

O4	Asp 16	O(H)*⋯*(O), O(H)*⋯*(O)	2.0, 1.8
	Gly 62	(O)H*⋯*N(H)	1.8
	Ser 63	(N)*⋯*N(H)	1.8
	Arg 131	(O)H*⋯*N(H), (O)H*⋯*N(H)	1.9, 2.0

O5	Asp 16	O(H)*⋯*(O), O(H)*⋯*(O)	1.8, 1.6
	Gly 62	(O)H*⋯*N(H)	1.6
	Ser 63	(N)*⋯*N(H)	1.8
	Arg 131	(O)H*⋯*N(H), (O)H*⋯*N(H)	2.0, 1.7

O6	Asp 16	O(H)*⋯*(O), O(H)*⋯*(O)	2.0, 1.9
	Gly 62	(O)H*⋯*N(H)	1.9
	Ser 63	(N)*⋯*N(H)	1.9
	Arg 131	(O)H*⋯*N(H)	2.1
	Asp 299	N(H)*⋯*(O)	2.1

N1	Asp 16	O(H)*⋯*(O)	1.9
	Thy 34	O(H)*⋯*(O), O(H)*⋯*(O)	2.0, 1.9
	Cys 37	O(H)*⋯*(O)	2.3
	Ser 63	(O)*⋯*N(H), (O)*⋯*N(H)	2.7, 1.9
	Arg 131	(O)*⋯*N(H), (O)H*⋯*N(H)	2.4, 2.2
	Ser 196	O(H)*⋯*(O)	2.2
	Asp 299	O(H)*⋯*(O)	1.7

N2	Asn 12	(O)*⋯*N(H)	1.8
